# ‘UltraGUD LP’—ultrasound guided diagnostic LP—a randomizedcontrolled trial. Traditional landmark based lumbar puncture is as effective as ultrasound guided lumbar puncture in outpatient neurology settings

**DOI:** 10.3389/fneur.2025.1521783

**Published:** 2025-03-10

**Authors:** Vijay Renga, Charlotte A. Jeffreys, Afsha Tabasum, Todd A. MacKenzie

**Affiliations:** ^1^Department of Neurology, Medical University of South Carolina, Charleston, SC, United States; ^2^Department of Neurology, Dartmouth Hitchcock Medical Center, Geisel School of Medicine at Dartmouth, Lebanon, NH, United States; ^3^Department of Biostatistics, Dartmouth Hitchcock Medical Center, Geisel School of Medicine at Dartmouth, Lebanon, NH, United States

**Keywords:** lumbar puncture, landmark LP, ultrasound LP, post LP headache, traumatic tap

## Abstract

**Background:**

Lumbar puncture (LP) is a fundamental procedure in neurology, yet its success is influenced by patient anatomy and provider expertise. Ultrasound guidance has been shown to improve outcomes in emergency and anesthesia settings, but its effectiveness in outpatient neurology remains unclear.

**Objective:**

This study (UltraGUD LP) aimed to compare the effectiveness of ultrasound-guided LP (US-LP) versus landmark-based LP (LM-LP) in an outpatient neurology setting, performed by a single experienced provider.

**Methods:**

A prospective randomized controlled trial was conducted from 2017 to 2022. Patients requiring LP were randomized to either LM-LP or US-LP. Success was defined as obtaining cerebrospinal fluid (CSF) within three attempts. Secondary outcomes included procedure time, incidence of traumatic taps, and post-LP headache rates.

**Results:**

Both techniques had comparable success rates, with LM-LP achieving 91% and US-LP 100% (*p* > 0.05). Procedure time was significantly shorter for LM-LP (13 vs. 19 min, *p* < 0.05). The incidence of traumatic taps and post-LP headaches was similar between groups.

**Conclusion:**

In a general outpatient neurology population, LM-LP is as effective as US-LP and requires less time. While US-LP may be beneficial for high-risk patients (e.g., obesity, prior back surgery), further studies are needed to confirm its superiority in these populations.

## Introduction

Lumbar puncture (LP) is an essential bedside procedure for diagnosing various neurological conditions. It is commonly performed in both outpatient and inpatient neurology settings. Due to the frequency with which neurologists perform this procedure, they are considered experts in LP. Other specialties often consult neurology to perform LPs.

However, bedside LP can be challenging due to patient factors such as advanced age, obesity, and prior back surgery. Uncertainty in bedside success and over-reliance on radiologic guidance have increased healthcare and personnel costs associated with this procedure.

The ultrasound-guided approach for lumbar puncture has demonstrated superiority in certain populations and is increasingly utilized. However, research exploring the advantages of ultrasound-guided lumbar puncture (US-LP) over traditional landmark-guided lumbar puncture (LM-LP) in routine adult neurology settings remains limited. To address this gap, our study (Ultra GUD LP – Ultrasound Guided Diagnostic Lumbar Puncture in Neurology) aims to assess the effectiveness of US-LP in an adult neurology setting.

The efficacy of ultrasound guidance for lumbar puncture (LP) remains controversial. While some studies have demonstrated increased success rates and reduced complications with ultrasound guidance (1–4), others have found no significant benefit over traditional landmark-based techniques (5–8). This variability in outcomes may be attributed to factors such as the clinical setting (e.g., emergency department, pediatric population, anesthesia), operator experience, and procedure techniques employed.

While generally safe, lumbar puncture (LP) can cause back pain, post-LP headaches, traumatic tap, or failure to obtain cerebrospinal fluid (CSF). These common side effects are usually transient. Due to the invasive nature of the procedure and the risk of rare but serious complications, patients may hesitate to undergo LP, fearing spinal cord damage, infection, and paralysis. Neurological complications from underlying disease may be misattributed to the LP. The success and complication rates of LP are influenced by patient factors (body habitus, age, history of back surgery, BMI, obesity, spinal deformities), provider experience, patient positioning, and procedural preparation.

Traditional landmark based lumbar puncture is a blind procedure that requires clinicians to identify the intersection of an imaginary Tuffier’s connecting both iliac crests and midline interspinous space (9, 10). This corresponds to the L4/5 space of the spinal canal. The performer feels the space and marks it on the skin. After disinfecting and anesthetizing, we insert a lumbar puncture needle into the interspace pointing toward the umbilicus that would typically reach the subarachnoid space and CSF is thus obtained. While the technique appears easy, providers often find it difficult to identify the interspace when the patient is obese or has spinal deformities or prior back surgeries. Bone or ligaments may often obstruct the needle and, despite multiple attempts, may not reach the desired location or injure nearby blood vessels, resulting in a bloody tap or hit nerves causing radicular pains. Up to 30% of landmark guided approaches could be misdirected with an overall success rate of 70–80% (8, 11). Meta analysis has shown a 90–95% success rate for ultrasound- guided LP compared to that of landmark-based methods (12). If bedside LP fails, patients often need to undergo a radiology guided procedure, which makes it significantly more expensive because of the need for additional personnel and technology. Transporting critically ill patients from ICU to a less equipped radiology suite can also pose a threat to the safety of a patient in case of any procedure-related or inherent complications. In addition to cost consideration, it also exposes the patient to radiation.

In the ultrasound guided technique, the L3/4 or L4/5 interspace is visualized using an ultrasound probe and the site of entry marked. Theoretically, it provides a more precise localization and a higher success rate. It would provide visualization in a patient where the traditional method is limited by inability to feel the interspace because of obesity, age-related or structural issues of spine or postoperative scarring. Since we can do ultrasound at bedside, it avoids the need to transport vulnerable patients to the radiology suite.

## Materials and methods

In the UltraGUD LP study, we attempted to study the effectiveness of Ultrasound-guided LP (US-LP) against Traditional Landmark Based LP (LM-LP) in the outpatient neurology clinics.

### Outcome measures

Primary endpoint was success in obtaining CSF. We defined success as the ability to get CSF within 3 attempts of needle reinsertion. Based on power analysis using a 30% failure rate for landmark based LP and 5% for Ultrasound guided LP a total of 72 subjects could have 80% power to detect anticipated differences between the group’s failure rate assuming two-sided hypothesis testing and an alpha level of 0.05.

Secondary measures included time taken, incidence of traumatic LP, post procedure pain, incidence of post LP headaches. Traumatic LP was defined as more than 400 RBCs per high-power field in the 4th test tube of CSF collected.

#### High risk of failure subjects

In order to determine effectiveness in difficult LPs we assigned a high risk population as subjects over the age of 50 or history of prior back surgery or BMI greater than 35.

#### Inclusion/exclusion criteria

Subjects above the age of 18 with an indication for a lumbar puncture for their neurologic evaluation were included in the study. Subjects who could not undergo a routine lumbar procedure due to anticoagulation, those who were unable to provide consent, or have allergy to latex, iodine or ultrasound gel used for the procedure were excluded.

### Implementation

This was a prospective, randomized controlled trial conducted between 2017 and 2022 at an outpatient neurology clinic. The study was approved by the Institutional Review Board and registered on ClinicalTrials.gov (NCT03815045).

All study participants were enrolled from the author’s institution. Written informed consent was obtained from all subjects prior to the study. Prior to the procedure, subjects were screened to exclude those with anticoagulant use within 72 h of the procedure and those with latex, ultrasound gel, chlorhexidine or iodine allergy.

Using a stratified randomization in RedCap subjects were randomized to either ‘Traditional-Landmark’ based (LM-LP) or ‘Ultrasound-Guided’ lumbar puncture (US-LP) group. Stratified randomization using REDCap is a technique that ensures balanced allocation of participants across predefined strata. REDCap’s randomization module allowed us to configure stratification factors and randomization sequences to minimize confounding variables and improve the reliability of results. We used age group (less than or greater than 50), gender (Male/Female), BMI (<35, >35), history of prior back surgery (yes/no), by integrating stratified randomization directly into REDCap, we were able to automate participant assignment in real-time while maintaining rigorous control over allocation criteria, ensuring a fair and unbiased distribution of participants across study arms. At the same time, this created an issue of insufficient slots. We created a 200 slot excel table for randomization initially which filled up early and therefore had to reinsert a table with over 2000 slots to allow for a higher redundancy needed for stratification. This caused more subjects to fall into the traditional landmark category compared to the ultrasound category because of the renewed randomization table.

The technique for ultrasound-guided lumbar puncture involves using ultrasound to identify and mark the optimal intervertebral space for needle insertion, typically between L3–L4 or L4–L5. The patients were positioned in sitting posture with a curved spine to maximize interspinous space exposure. A high-frequency linear probe is placed longitudinally along the midline process. This visualizes the hyperechoic spinous process as mounds between which the hypoechoic interspinous space exits through which needle is passed. This interspinous space is marked on both sides of the transducer and creates a horizontal line. The transducer is then rotated into a transverse orientation which helps confirm the interspinous location. Moving the transducer up and down helps detect and mark the spinous process above and below. The line joining these points form a vertical line along the midline. The point of intersection of a horizontal and vertical line is marked as the site of needle entry. Key landmarks, including the spinous processes, interspinous space, ligamentum flavum, and dural sac, are visualized on the ultrasound. After identifying the ideal trajectory, the skin is sterilized, and a spinal needle is inserted. The needle tip is advanced carefully, piercing through the skin, subcutaneous tissue, supraspinous and interspinous ligaments, and ligamentum flavum before entering the subarachnoid space for cerebrospinal fluid collection.

The primary author who performed all LPs had over 20 years of experience in performing traditional lumbar puncture and over 2 years of experience with an ultrasound-guided technique. All subjects underwent lumbar puncture done by the author using regular LP needles in an upright sitting and leaning forward position except two subjects in whom CSF opening pressure was required and therefore performed in the left lateral decubitus position. Secondary measures included the time taken for the procedure and post LP complications, including back pain, headache, traumatic tap. We timed each procedure from the localization using palpation or using ultrasound to the time CSF was obtained (in minutes). We assessed post procedure pain at 5 min after the procedure. Occurrence of post LP headaches or other complications was assessed at 72 h with a follow-up phone call.

We enrolled 38 subjects before an interim analysis. We excluded two subjects due to randomization error, and we aborted one procedure because of vasovagal syncope at the time of needle insertion. Study was completed in 35 subjects. Twenty-two subjects underwent landmark-based lumbar puncture, and 13 subjects underwent ultrasound- guided procedure. Subjects were prepped and positioned in a standard manner. Time taken from positioning to completion was calculated as procedure time. This included ultrasound guided localization, skin preparation, local anesthesia and needle insertion, to getting CSF or failing to get within 3 attempts. In subjects needing ultrasound guidance, a point of care ultrasound probe (Butterly IQ) was used to localize interspinous space using a horizontal orientation for midline identification and vertical orientation for interspinous space identification. No significant procedure-related complications occurred during the study. One subject had vasovagal syncope where the procedure was aborted. Even though we planned to enroll 72 subjects, after this interim analysis at the halfway point, we concluded the study.

Statistical analysis was performed to study the effectiveness of UltraGUD LP on the following binary patient primary outcomes: obtaining CSF within 3 attempts, RBC count >400, and occurrence of post-procedure headache. Due to low sample sizes we conducted a Fisher’s Exact test to test for a statistically significant association between lumbar puncture procedure type and occurrence of post-procedure headache. Cohen’s h was used to measure the effect size of success rate differences between groups. We also studied the effects of UltraGUD LP on the following secondary outcomes: procedure time (minutes), number of attempts needed (1–3 attempts), and success rates for high-risk patients using the Wilcoxon Rank test.

## Results

### Baseline characteristics

Thirty-five patients completed the study (22 in LM-LP, 13 in US-LP). Groups were comparable in age (mean: 53 vs. 57 years), BMI (mean: 28 for both), and high-risk factors (BMI >35, prior back surgery, age > 50). There were 17 high risk subjects in the traditional group and 10 in the ultrasound group. The Chi-Square Test Results for Baseline Differences in high risk groups also indicated that there were no statistically significant differences between the intervention groups (ultrasound vs. traditional) in terms of BMI >35 (*p* = 0.34), prior back surgery (*p* = 0.50), age > 50 (*p* = 0.81), or sex (*p* = 0.81).

### Primary outcome

- LM-LP success rate: 91% (20/22)
- US-LP success rate: 100% (13/13)
- The difference was not statistically significant (*p* > 0.05).

### Secondary outcomes

- Procedure time: LM-LP: 13 min vs. US-LP: 19 min (*p* < 0.05).
- Traumatic tap incidence: LM-LP: 9% (2/22) vs. US-LP: 7.7% (1/13) (*p* > 0.05).
- Post-LP headaches: LM-LP: 13.6% (3/22) vs. US-LP: 23.1% (3/13) (*p* > 0.05).

Of the subjects who failed using LM-LP, one had a history of multiple back surgeries and chronic arachnoiditis who also had a failed CSF attempt with radiology guidance and the other patient had DISH (Diffuse Idiopathic Skeletal Hyperostosis). The 95% confidence interval for success in the traditional group ranged from 72.9 to 100%. Average number of attempts was 1.6 in the landmark group versus 1.5 in the ultrasound group. Three subjects in each group developed post LP headaches. Post-LP headaches occurred slightly more often in the ultrasound group (23.1% vs. 13.6%), though this was not statistically significant. RBC count >400 occurred in 2 patients in traditional and one in ultrasound group. Fisher’s exact test found no significant association between procedure type and post-LP headache or RBC count.

### High-risk subgroup analysis

**Figure 1:**
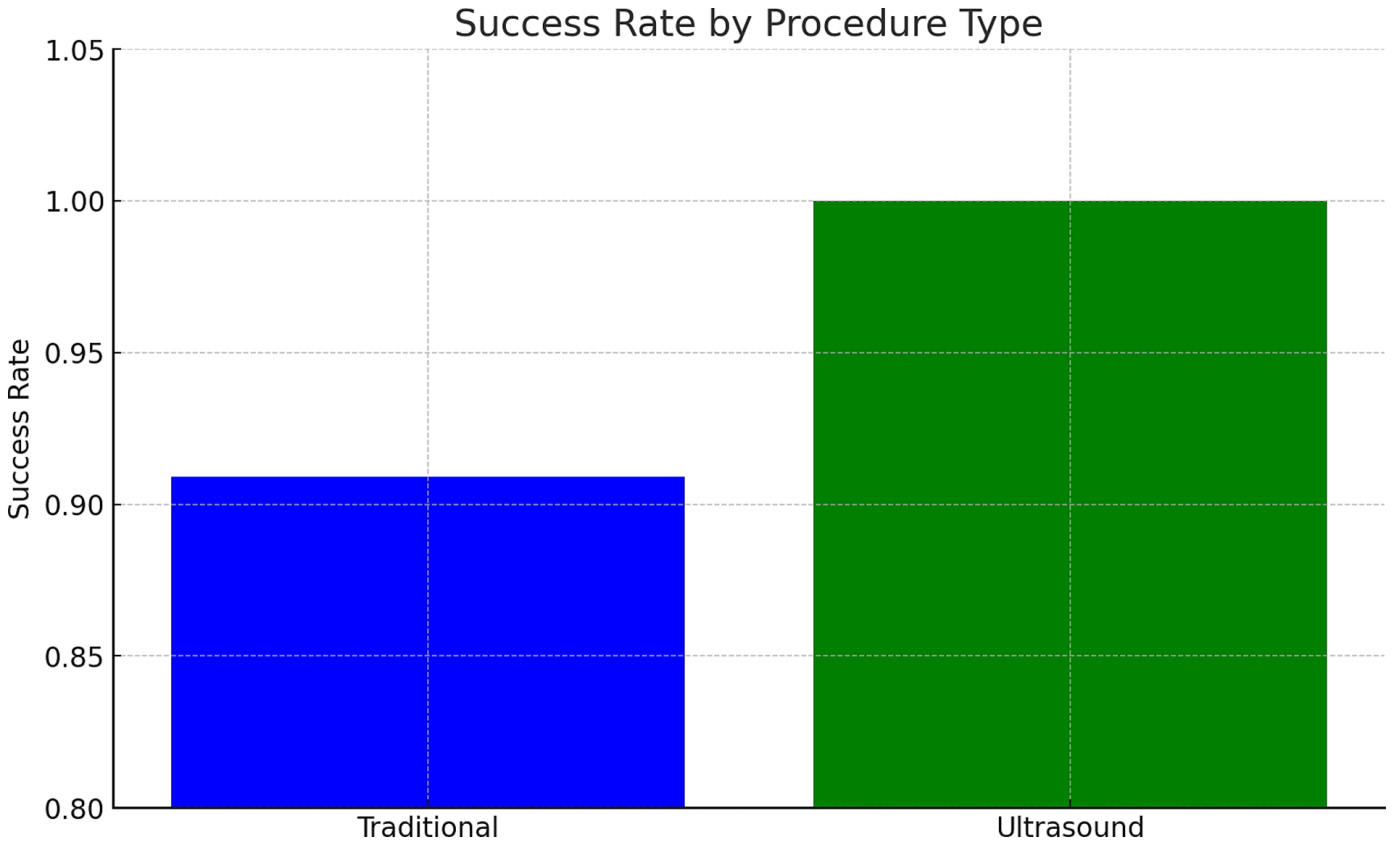
Procedure success rate by procedure type – overall.

**Figure 2:**
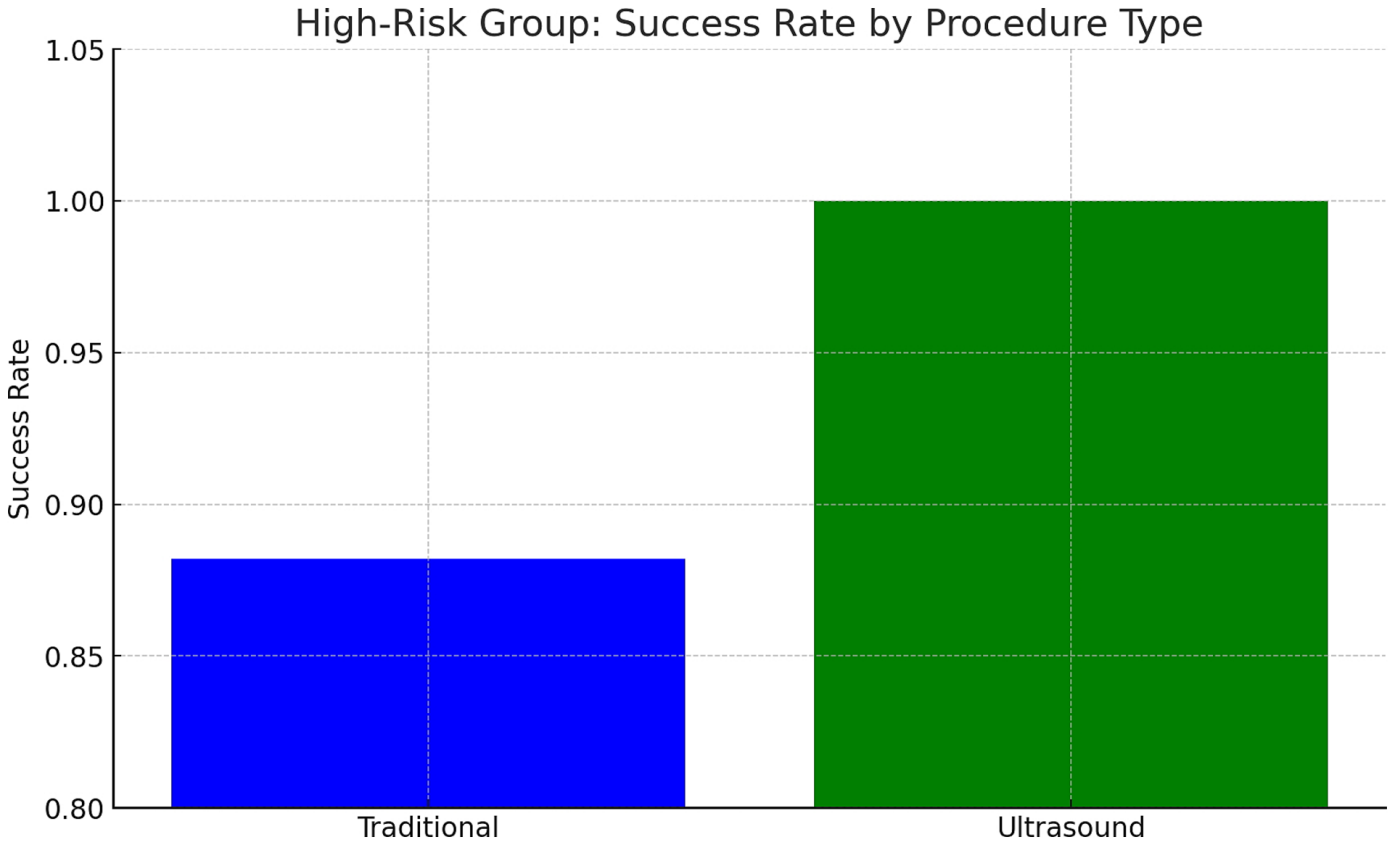
Procedure success in high-risk subjects.

**Figure 3:**
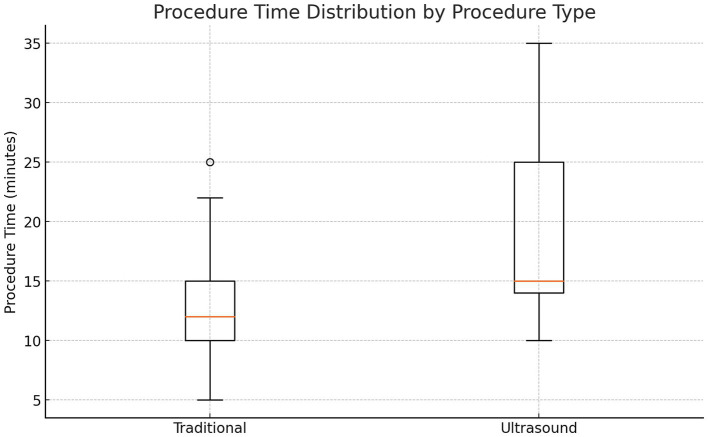
Procedure time taken overall.

**Figure 4:**
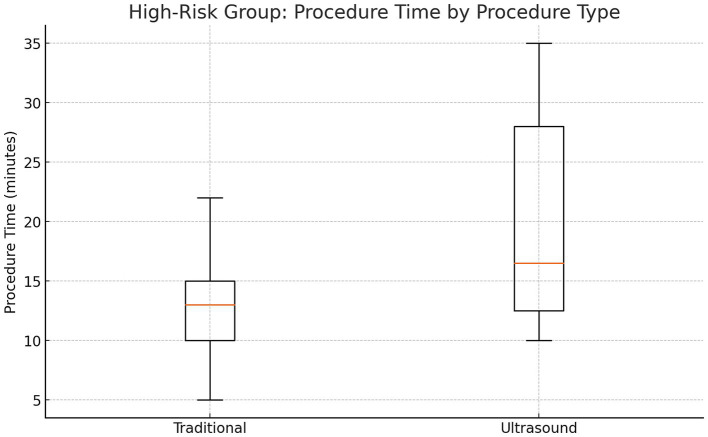
Procedure time in high-risk subjects.

In patients with BMI >35, prior back surgery, or age > 50, US-LP had a higher success rate (100% vs. 88.2%) (Cohen’s *h* = 0.70) (*p* = 0.71). US-LP in high risk group had fewer post-LP headaches (0% vs. 17.6%), though differences were not statistically significant. However, US-LP required significantly more time (20 vs. 13 min, *p* = 0.042). A more detailed analysis of procedural inefficiencies in high risk group revealed that while ultrasound improves success rates, it does not necessarily reduce the number of attempts (mean attempts: 1.5 vs. 1.65, *p* = 0.61, Cohen’s *d* = −0.21). However, ultrasound significantly increased procedure time (20.0 vs. 13.0 min, *p* = 0.042, Cohen’s *d* = 0.96, large effect size), indicating a potential trade-off between accuracy and efficiency (Figures 1–4 and Table 1).

**Table 1:** Procedure type, patient characteristics and outcomes in overall and high-risk groups.

	Overall population					High risk group					Metrics		
Procedure_Type	Subjects	Mean_Age	Mean_BMI	Successful_LPs	Success_Percentage	Age > 50	BMI > 35	Prior_Back_Surgery	High risk	Success_Percentage HighRisk	Avg_Time_Taken	Post_LP_Headache	RBC_Count > 400
Traditional	22	53	28	20	90.9%	13	6	7	17	88.2%	12.7	3	2
Ultrasound	13	57	28	13	100.0%	9	1	2	10	100%	18.8	3	1

## Discussion

This study found no significant advantage of US-LP over LM-LP in an outpatient neurology setting when performed by an experienced provider. LM-LP was faster, making it preferable for routine cases. US-LP may be beneficial in high-risk patients with obesity, spinal deformities, or prior surgeries but requires further validation. The high LM-LP success rate in this study (91%) exceeds previously reported rates (70–80%). This may be due to standardization and the experience of the sole provider, reducing variability. In prior studies, LPs were performed by providers with varying expertise, potentially affecting outcomes. Although ultrasound guidance enhances anatomical visualization, it increases procedural time due to setup and marking requirements. The decision to use ultrasound should weigh these factors, particularly in busy clinical settings.

Beyond the statistical findings, it is essential to highlight ultrasound guidance’s mechanistic advantages, including real-time visualization of the interspinous space, measurement of needle depth, and avoidance of vascular structures. These benefits may explain the observed trends toward higher success rates and fewer complications in high-risk patients. Particularly in obese individuals and those with spinal deformities, where traditional palpation is unreliable, ultrasound offers a significant advantage by allowing precise anatomical localization despite excessive adipose tissue or post-surgical changes. Our findings support using ultrasound in these complex cases, even though statistical significance was not reached. While statistical significance was not achieved in all comparisons, the observed trends suggest meaningful advantages for high-risk populations.

### Strengths

Compared to prior studies, our study minimized variability by having a single provider performing all LPs using the same technique.

### Limitations

This study used a small sample size, and we concluded the study after an interim analysis showing insignificant differences in the regular adult population. The author having more experience with traditional landmark-based LP than ultrasound- guided LP could have impacted the time taken. This study focused on a general outpatient neurology population which may differ from inpatient settings in terms of procedure settings and patient conditions.

Comparison of US-LP to LM-LP in high-risk populations showed a clear trend toward higher success rates, which could have reached significance levels had the sample size been higher. A more focused study that compares LM-LP vs. US-LP only in at-risk-of-failure subjects with BMI >35, prior back surgery and skeletal deformities could have provided more information regarding the superiority of the ultrasound-guided technique. A recent study looking at high BMI patients in neurology has shown a significantly higher success rate of 93% vs. 68% and a shorter duration of 17 min vs. 37 min than the conventional LP group (13). The average time taken for landmark-based LP in this study done by trainees was thrice as much as that of our study, whereas the ultrasound-guided procedure had a similar duration to our research.

The Society of Hospital Medicine advocates using Ultrasound Guidance whenever possible to improve success rates and minimize attempts for procedures like lumbar punctures (14). However, the additional time and resources required for ultrasound-guided procedures must be balanced against the demands of a busy hospital environment. As evidenced by this and previous studies, ultrasound may be most beneficial for patients who have failed traditional methods or who are at risk of failure due to obesity, positioning difficulties, or prior back surgeries.

Ultrasound guidance requires specialized training, equipment, and resources, and its use can extend procedure times. For example, ultrasound-guided skin markings can shift with patient movement, necessitating re-marking after repeating aseptic precautions. Promising new techniques, such as real-time ultrasound in diagnostic lumbar puncture and computerized training programs of abnormal spine models, may improve procedural success rates (15, 16). While these techniques may require additional training to master, they could address the issue of site markings shifting during the procedure.

Although ultrasound can enhance procedural accuracy, its widespread adoption is contingent upon factors such as provider expertise, equipment availability, and time constraints. Ultrasound-guided techniques necessitate substantial training and hands-on experience, which may impede their implementation in busy clinical settings. Future research should prioritize optimizing ultrasound techniques, evaluating real-time ultrasound guidance, and assessing its cost-effectiveness in neurology.

The results of this study may not be generalizable to trainees or less experienced providers, given the variability in LM-LP results based on provider experience. In fact, an ultrasound approach could improve success rates for these groups. Additionally, the author noted a personal observation of increased success rate with traditional LPs during this study compared to prior experience. This improvement can be attributed to the standardized LP settings maintained consistently as part of the research project, which reduced variability and increased success rates. Therefore, an important component of a successful lumbar puncture is the attention and focus applied to the procedure, in addition to an optimized and consistent procedural setting.

## Conclusion

LM-LP remains a highly effective and efficient technique in outpatient neurology. US-LP offers potential benefits for high-risk populations but requires further study. Given the additional time and resource requirements, routine ultrasound use may not be justified for all patients.

## Data Availability

The raw data supporting the conclusions of this article will be made available by the authors, without undue reservation.
